# Genome-Wide Identification of Copy Number Variations in Chinese Holstein

**DOI:** 10.1371/journal.pone.0048732

**Published:** 2012-11-07

**Authors:** Li Jiang, Jicai Jiang, Jiying Wang, Xiangdong Ding, Jianfeng Liu, Qin Zhang

**Affiliations:** Key Laboratory of Animal Genetics, Breeding and Reproduction, Ministry of Agriculture, College of Animal Science and Technology, China Agricultural University, Beijing, People’s Republic of China; The Roslin Institute, University of Edinburgh, United Kingdom

## Abstract

Recent studies of mammalian genomes have uncovered the vast extent of copy number variations (CNVs) that contribute to phenotypic diversity. Compared to SNP, a CNV can cover a wider chromosome region, which may potentially incur substantial sequence changes and induce more significant effects on phenotypes. CNV has been becoming an alternative promising genetic marker in the field of genetic analyses. Here we firstly report an account of CNV regions in the cattle genome in Chinese Holstein population. The Illumina Bovine SNP50K Beadchips were used for screening 2047 Holstein individuals. Three different programes (PennCNV, cnvPartition and GADA) were implemented to detect potential CNVs. After a strict CNV calling pipeline, a total of 99 CNV regions were identified in cattle genome. These CNV regions cover 23.24 Mb in total with an average size of 151.69 Kb. 52 out of these CNV regions have frequencies of above 1%. 51 out of these CNV regions completely or partially overlap with 138 cattle genes, which are significantly enriched for specific biological functions, such as signaling pathway, sensory perception response and cellular processes. The results provide valuable information for constructing a more comprehensive CNV map in the cattle genome and offer an important resource for investigation of genome structure and genomic variation underlying traits of interest in cattle.

## Introduction

With the rapid progress of genome sequencing, the abundance of single nucleotide polymorphisms (SNPs) as a major source of genetic variation has been widely recognized. As a result, great efforts were made to develop high-throughput SNP genotyping platforms, and genome-wide high density SNP chips have been designed for many species including human and major farm animal species, such as cattle, swine, sheep, and chicken. Using these SNP chips, a large number of genome-wide association studies (GWAS) have been carried out in milking and meat production as well as diseases in cattle [1], [2], [3]. Over last few years, a few of crucial SNPs have been identified and confirmed with effects on milk production traits, *e.g.*, the K232A mutation in DGAT1 [4].

In addition to SNPs, another form of genetic variation, *i.e.*, copy number variation (CNV), has been identified in many species, including human [5], [6], [7], [8], [9], [10], mouse [11], [12], [13], [14], [15], rat [16], fruit fly [17], [18], dog [19], pig [20], [21], and cattle [22], [23], [24], [25], [26]. CNV is defined as a variable copy number of DNA segments ranging from 1 kilobase (Kb) to several megabases (Mb) compared with a reference genome [27]. CNVs take several forms, including deletions, duplications, insertions and complex rearrangements in the genome. So far, there are 179,450 CNVs identified in human genome which cover more than 53% of the human genome according to the Database of Genomic Variants (DGV) (http://dgvbeta.tcag,ca/dgv/app/home?ref=NCBI36/hg18, Apr, 2012). Thousands of genes, regulation elements and segmental duplications are harbored within these CNV regions [27], [28]. CNVs can potentially influence phenotypes or lead to diseases by altering gene dosage and/or disrupting genes in the form of deletion or duplication [29], [30], [31]. Furthermore, CNVs can modulate gene expression indirectly through disturbing the regulation regions of genes [5]. It has been found that many CNVs contribute to phenotypic variation in animals [32], [33], [34] as well as in humans [35], [36], [37], [38], [39].

Currently, CNVs can been identified using different technological approaches. Two major platforms are commonly used. One is the comparative genomic hybridization (CGH) array based approach [9], [25], [40], [41], [42], in which signal intensities of reference and target DNA samples labeled with different fluorescent tags are compared. The other is the SNP array based approach [24], [43], [44], in which intensity values of SNPs derived from each sample are used to estimate copy numbers in each individual. In comparison between these two existing panels, CGH array based approach has excellent performance in signal-to-noise ratios, while the SNP array based approach is more convenient for high-throughput analyses and follow-up association studies [45]. With the development of high density SNP arrays, higher resolution of genomic regions can be achieved [46]. Furthermore, recent advances in next-generation sequencing technology allow for more detailed characterization of CNVs [47], [48], [49], [50] and detect CNVs with a higher effective resolution and sensitivity and become more and more popular due to the cost decreases for sequencing. Therefore, many studies pay more attention to efficient algorithms to detect reliable CNVs via SNP array data [51], [52] and sequence data [53], [54].

So far, only a few CNV studies in cattle have been performed and relative few CNVs were detected or confirmed. Using CGH array, Fadista et al. [23] reported 304 CNV regions (CNVRs) from 20 bovine samples derived from 4 dairy and beef breeds, and Liu et al. [25] identified over 200 CNVRs from 90 animals of several different cattle breeds. By SNP array, Bae et al. [22] identified 368 CNVRs from 265 samples, Hou et al. [24], [26] reported 682 candidate CNVRs in 521 animals of 21 cattle breeds and 811 CNVRs in 472 Angus cattle. Using sequencing platform, Bickhart et al. [48], Zhan et al. [49] and Stothard et al. [50] reported 1265, 520 and 790 CNVRs from one, two and five individuals, respectively. Although some novel CNVRs were found by sequencing platform in these studies, it is limited by using very limited numbers of tested animals. Compared with the coverage of CNVRs detected in the human genome, the total length of CNVRs reported in cattle only cover 0.13% (3.3 Mb) to 5.57% (141.8 Mb) of the cattle genome. It can be envisaged that there are still a large number of CNVs undetected. Considering potential significance of CNV contributing to complex traits, further efforts should be made to obtain a more comprehensive CNV map in cattle genome.

In this study, we investigated genome-wide CNVRs in a Chinese Holstein population with a larger sample size of 2047 individuals. To pursue convincing results, we employed three programs (PennCNV, GADA and cnvPartition) based on different algorithms to analyze Bovine SNP50 genotyping data along with very strict quality control. Consequentially, we identified 99 candidate CNV regions. Our study provides useful addition to the cattle CNV map.

## Materials and Methods

### Sample Collection and Genotyping

The study population consisted of 2047 Chinese Holstein cattle, including 1960 cows and 87 sires (of which 14 are sires of these cows, each has 83 to 358 daughters) with unknown relationship. The Chinese Holstein originated from crosses of European Holstein-Friesian with Chinese Yellow cattle about 70 yr ago. Since then, continuous introgression of foreign Holstein genes (live bulls, semen, and embryos), mainly from North America, have been conducted. Therefore, the current population has a close relationship with the North American Holstein.

DNA was extracted from blood samples of cows and semen samples of bulls. The concentration and the purity of genomic DNA were assessed on the Nanovue Spectrophotometer. All samples were genotyped with the Illumina BovineSNP50 BeadChip. All the markers were clustered and genotyped using the BEADSTUDIO software.

The blood samples were collected along with the regular quarantine inspection of the farms, so no ethical approval was required for this study.

### CNV Detection

In order to increase the confidence in CNV detection and decrease the rate of false discoveries, we used three programs to infer CNVs: PennCNV [55], cnvPartition v2.4.4 Plug-in (http://www.illumina.com) and GADA (Genome Alteration Detection Algorithm, [56]). The required data on signal intensities (Log R ratio, LRR) and allelic intensity ratios (B allele frequency, BAF) of all SNPs for all samples were generated from the Illumina BeadStudio 3.5 software (Illumina). For PennCNV, which is the most widely used program for inferring CNV based on SNP data [21], [24], the analysis of the X chromosome and autosomes were separately performed. PennCNV was run using the *–test* option without considering pedigree information since the cows in our study population merely have sire information and the relationship of these bulls is unknown. The PFB (population frequency of B allele) file was generated based on the BAF of each marker in this population. The signal intensity of each SNP which is subject to genomic waves was adjusted for the GC content of the 500 Kb genomic region of its both sides. The parameters involved were defined as 0.24 for standard deviation of LRR, 0.01 for BAF_DRIFT and 0.05 for waviness factor. For cnvPartition v2.4.4 Plug-in, the default parameters set by Illumina were used. For GADA, the parameters involved were defined as 0.8 for sparseness hypeparameter (a_α_) and 8 for critical value of the backward elimination (BE).

For each program, we employed the following criteria to define a potential CNV: its size was less than 1 Mb; it contained three or more consecutive SNPs; and it was detected in at least two animals (the overlapped region detected in different animals was defined as a CNV). In addition, to minimize the false positive rate, the union region of overlapping CNVs detected by different programs was defined as a CNV region (CNVR).

Information on gene annotations within the CNVRs was retrieved from the NCBI Gene Database based on Btau_4.0 genome assembly (The Bovine Genome Sequencing and Analysis Consortium, 2009).

### qPCR Validation

Quantitative real time PCR (qPCR) was used to validate CNVRs or CNVs detected in the study. The relative comparative threshold cycle (2^−△△C^T) method was used to quantify copy number changes by comparing the ΔC_T_ [cycle threshold (C_T_) of target region minus C_T_ of control region] value of samples with CNV to the ΔC_T_ of a calibrator without CNV [57], [58], [59]. CNVRs (CNVs) were tested by using SYBR Green chemistry as recommended by the manufacturers. We designed the PCR primers using Primer 3 web tool (http://frodo.wi.mit.edu/primer3/). For each target CNVR, two pairs of primers were designed considering the uncertainty of the CNV boundaries. Moreover, In-Silico PCR program from the UCSC browser (http://genome.ucsc.edu/) was used for *in silico* specificity analysis to ensure the primers only matching with the sequence of interest. We generated standard curves for each primer of target and control regions in order to ensure approximately equal PCR efficiencies between them. A serial diluted genomic DNA samples from a common cattle was used as template for creating a standard curve of each primer. Amplification efficiencies of all primers were calculated based on the standard curves. The copy number of each CNVR (CNV) was compared with a region in the control gene *Basic transcription factor* 3 (*BTF3*) as done in previous studies [22]. All PCR primers were designed based on its reference sequence in NCBI. PCR amplifications were performed in a total volume of 20 µL consisting of the following reagents: 1 µL DNA (around 50 ng), 1 µL(20 pM/µL) of both forward primer and reverse primer, 10 µL of Master Mix (2×) and water (Roche Applied Science). All RT-PCRs were run in triplicate. PCRs were run as follows: 5 min at 95°C followed by 40 cycles at 95°C for 10sec and 60°C for 10 sec. All PCRs were performed in 96-well clear reaction plates (Roche Applied Science). The average C_T_ value of three replications for each sample was calculated and normalized against the control gene with the assumption of existing two copies of DNA segment in the control region. For each CNVR (CNV) to be validated, a value from the formula 2×2^−△△C^T was calculated for each individual. The value obtained was used to judge if an individual is in normal status without CNV (if the value was around 2), in gain status (if the value was around 3 or above), or in loss status (if the value was around 0 or 1).

## Results

**Figure 1:**
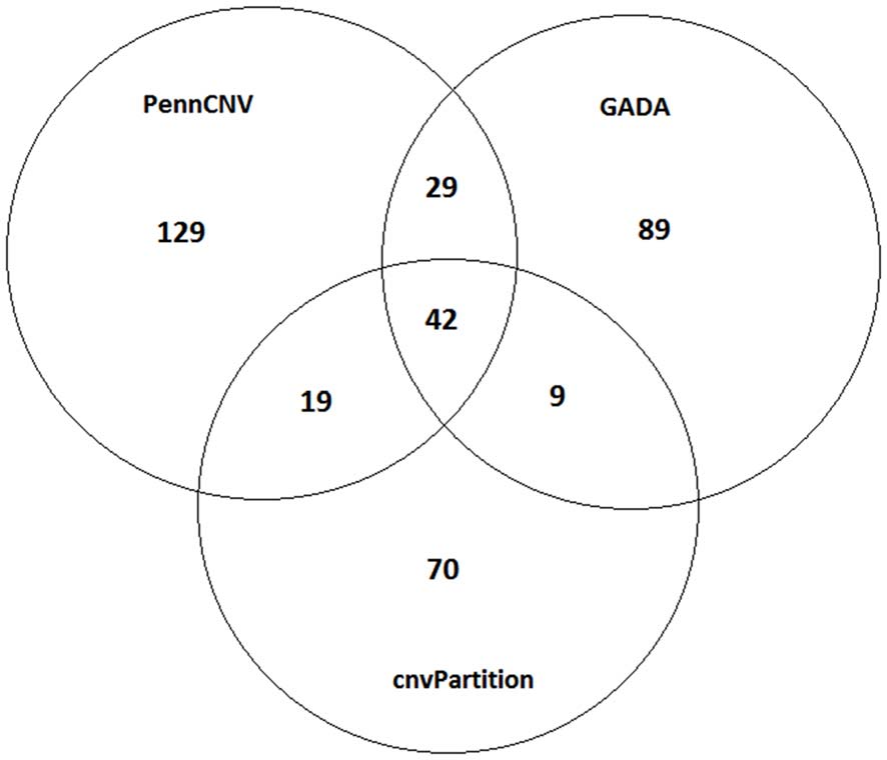
Numbers of CNVs identified by three programs and numbers of CNVs overlapped between different programs.

**Figure 2:**
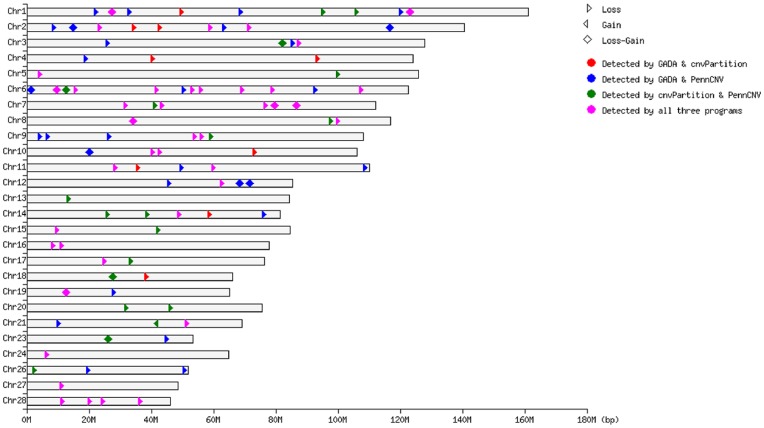
The distribution and status of detected CNVRs across the bovine genome (based on the Btau_4.0 assembly).

The numbers of CNVs called by PennCNV, GADA and cnvPartition were 219, 169 and 140, respectively (Fig. 1). Among these CNVs, 71 were commonly called by both PennCNV and GADA, 61 by both PennCNV and cnvPartition, 51 by both GADA and cnvPartition, and 42 by all of the three programs. A total of 99 CNVRs (union region of overlapping CNVs called by two or three programs) across genome were identified. The lengths of these CNVRs range from 27.01 Kb to 1.31 Mb, with an average size of 234.76 Kb and a median size of 151.69 Kb. The total length of all CNVRs is 23.24 Mb and covers 0.91% of the whole bovine genome. These CNVRs are located on all chromosomes except BTAs 22, 25, 29 and X. The numbers of CNVRs vary across different chromosomes, with BTA6 having the largest proportion (Fig. 2). Among the 99 CNVRs, 81 are in loss status, one in gain status and 17 in loss-gain status. The frequencies of these CNVRs in the study population are quite different. Specifically, 14 (14.1%), 17 (17.2%), 22 (22.2%), 34 (34.3%) and 52 (52.5%) CNVRs have frequencies of above 5%, 4%, 3%, 2% and 1%, respectively. Furthermore, 11 CNVRs were identified in more than 100 individuals, 63 CNVRs in more than 10 animals and the rest in more than 3 individuals. The CNVR with the highest frequency is on BTA10, reaching 27.09% in the population. The detailed description of each CNVR identified is given in Table S1.

These identified CNVRs contain abundant annotated genes. A total of 138 genes are harbored within 51 CNVRs (Table S2), of which 20 contain two or more annotated genes. On the other hand, there are 48 CNVRs without any known genes.

**Figure 3:**
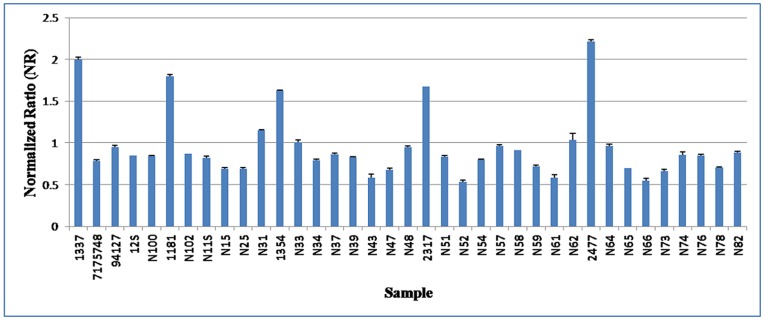
Results of qPCR validation for one CNVR (No. 43). NR around 2 indicates normal status (no CNV) and NR around 1 indicates one copy loss. The error bars represent the standard error among three technical replicates.

To further convince our results, we selected 6 CNVRs and 6 CNVs (detected only by PennCNV) to be validated by qPCR. These CNVRs or CNVs represent different status of copy numbers variation (i.e., loss and both) and different CNVR frequencies (varied from 0.19 to 6.3%) (See Table S1). In summary, of the 6 CNVRs, 4 (IDs = 43, 78, 80, and 84) were confirmed by qPCR, while of the 6 CNVs only 2 (CNV IDs = 100 and 101) were confirmed. Figure 3 illustrates the qPCR results for one confirmed CNVR (ID = 43). The full results for all of the 6 CNVRs are given in Figure S1. The detailed information of the validated CNVRs or CNVs and the primers used in qPCR analyses is given in Table S3.

## Discussion

CNVs contribute greatly to the genomic structure variation. In the past few years, CNVs have been explored extensively in the human genome and some of them were found to play important roles in disease susceptibility [52], [60], [61]. In animals, CNVs also contribute to the variation of phenotypes or some common diseases. For instance, the Pea-comb phenotype in chicken is caused by the duplication of the first intron of the *Sox5* gene [32]. The white coat phenotype in pigs is caused by the copy number variation of the *KIT* gene [10]. Copy number variation of the *ASIP* (agouti signaling protein) gene in goat leads to different coat colors [62]. It is also reported that CNVs may be associated with cattle health and adaptive traits [26], [34]. These demonstrate that CNVs can be considered as promising markers for some traits or diseases in domestic animals. Our results illustrate the widespread of CNVR in Chinese Holstein genome. In total, 387 CNVs were detected by either of the three programs (PennCNV, GADA and cnvPartition), 99 out of which were called by at least two programs.

So far, a number of algorithms have been developed to infer CNVs based on SNP data. In order to minimize the false positive rate, we used three programs to detect CNV. It can be found that different methods lead to different results. 219, 169 and 140 CNVs were detected by PennCNV, GADA, and cnvPartition, respectively, and only 42 were commonly detected by the three programs. The inconsistence between different programs was also reported in other CNV studies. For example, using data from Porcine SNP60 BeadChip and also the same three programs as we used here, Ramayo-Caldas et al. [21] reported 94, 84, and 200 CNVs called by cnvPartition, PennCNV and GADA, respectively, in pigs from an Iberian x Landrace cross and only 26 were overlapped among them. Winchester et al. [63] compared 7 programs (including PennCNV, GADA and cnvPartition) using a common data set from the HapMap collection and found a large variation in numbers of copy number events among these algorithms. The inconsistence among different programs should be mainly due to the different algorithms implemented in these programs. In particular, PennCNV is based on the Hidden Markov model, GADA uses sparse Bayesian learning algorithms, while cnvPartition is a plug-in software within BeadsStudio (illumina) which uses the log R ratio and BAF and compares the data to 14 different Gaussian distribution models to assess copy number level. Each algorithm has its strengths and weaknesses as summarized by Winchester et al [63]. Therefore, Winchester et al. [63] recommended using multiple algorithms on a single dataset to produce the most informative results and also utilize the different advantages of each software.

Although the Bovine 50 K Beadchip is feasible for CNV detection, SNP probes on the chip are neither dense enough nor uniformly distributed to achieve an unbiased and high-resolution cattle CNV map. The average interval between adjacent SNPs on the Bovine 50 K Beadchip is 51.5 Kb. In addition, this chip was originally developed for SNP genotyping in association studies, and a large proportion of probes may be positioned beyond CNVRs. Hence, only the CNVRs with sufficient length were expected to be discovered. Some studies in humans suggested that smaller CNVs are much more frequent than larger ones [10], [64]. With application of the Bovine high-density 800 K chip or next generation sequencing methods, it can be expected that CNV resources across genome can be increasingly identified.

It is notable that these 99 CNVRs include 81 loss, 1 gain and 17 both (loss and gain) events in our study, i.e., loss-type CNVs are much more common than gain-type ones. Similar results have been reported in other studies [22], [23], [24]. But this is different from the results reported in the human genome studies and in the porcine genome studies [21]. This may be because that some CNVRs are not discovered in our study due to the limitation of the Bovine SNP 50 K Beadchip and the strict quality control criteria.

CNV content varies significantly among different chromosomes. The proportion of the total CNVR length on different chromosomes to the length of the chromosome ranged from 0.19% to 3.90% (see Table S7). Chromosomes 6, 1 and 2 show the greatest enrichment of CNVRs with two-fold of the average CNVR content across the whole genome. Compared with the reported CNVRs of bovine genome based on SNP array [22], [23], [24], [25], [26], our results are largely consistent with them (see Table 1). Specifically, 70 CNVRs (70.7%) in our results are overlapped with those reported by Hou *et al.* [24] and the total length of overlapped regions is 12.2 Mb (52.6%), 42 CNVRs (42.4%) overlapped with those reported by Bae *et al.* [22] and the length of overlapped region is 5.4 Mb (23.3%). In comparison with the CNV findings based on CGH-array, only 11 CNVRs (11.1%) with the total length of 0.8 Mb (3.4%) and six CNVRs (6.1%) with the total length of 0.7 Mb (3%) identified in our study are overlapped with those reported by Fadista *et al.* [23] and Liu *et al.* [25], respectively. In addition, we compared our results with the CNVRs detected based on sequence data [48], [49], [50]. The number of overlapped CNVRs varies from 10 to 77. The total length of 0.2 Mb (9%), 0.1 Mb (5%) and 0.36 Mb (16%) in our study are overlapped with those reported by Bickhart et al. [48], Zhan et al. [49] and Stothard et al. [50], respectively (Table 1). This demonstrates that different technology platforms for genome-wide CNV surveys can lead to different results, and it also illustrates that even using the same platform and program, different sets of CNVs can be inferred in different populations due to differences in population genetic background, sample size, CNV and CNVR definition, and technical errors. Since the identified CNVRs by different studies do not completely overlap, a great amount of CNVRs are still undiscovered in cattle genomes.

**Table 1:** Comparison between results of the current study and results from other studies.

	Findings from different studies				Overlapped CNVRs with this Study		
	Study	Count	Total Length(Mb)	Count	Percentage ofcount	Total length(Mb)	Percentage oflength
CGH-based Studies	Fadista et al. [23] a	266	16.6	11	11.1%	0.8	3.4%
	Liu et al. [25] b	177	28.1	6	6.1%	0.7	3%
							
SNP-based Studies	Hou et al. [24]	682	139.8	70	70.7%	12.2	52.6%
	Bae et al. [22]	368	63.1	42	42.4%	5.4	23.3%
	Hou et al. [26]	811	141.8	59	59.6%	10.6	45.7%
							
Resequencing-based	Bickhart et al. [48]	1265	55.6	10	10.1%	0.202	0.9%
studies	Zhan et al. [49]	520	3.6	16	16.2%	0.112	0.5%
	Stothard et al. [50]	790	3.3	77	77.8%	0.367	1.6%
							
This study		99	23.2				

a : CNVRs on Chr Un and mitochondrial sequence are excluded;

b : CNVRs on Chr Un are excluded.

Previous studies have shown that CNVs play an important role in phenotypic variation and are often related with disease susceptibility [5], [65], [66]. We compared the 99 CNVRs identified in this study with the reported QTL regions collected in the cattle QTL database (http://www.animalgenome.org/cgi-bin/QTLdb/BT/index). Since some QTLs have too large confidence interval and some QTLs reported by different studies are overlapped, we focused on QTL with confidence interval less than 30cM and considered those QTLs with overlapped confidence intervals greater than 50% as the same QTL. In this way, we identified 402 QTLs in total. 95 out of the 99 CNVRs harbor or partially overlap with 69 (17%) such QTL (Table S4). Since the total length of the 99 CNVRs covers only 0.91% of the whole bovine genome, there is a much greater QTL density coinciding with the CNVRs than we see in the genome as a whole. These QTLs are involved in many disease susceptibility traits, such as clinical mastitis, somatic cell score, bovine spongiform encephalopathy and gastrointestinal nematode burden (see Table S4). There are also CNVRs harboring QTLs which are associated with feed conversion, milk production and reproduction traits, such as calving ease, gestation length, birth body weight and non-return rate (Table S4). We also performed highly conserved elements (HECs) analysis and found 5,660 conserved elements in the CNVRs. The number of HECs in each CNVR is given in Table S5.

Furthermore, 51 of the identified CNVRs are completely or partially overlap with regions of bovine genes and encompass 138 known genes in total. The DAVID Bioinformatics Resources v6.7 [http://david.abcc.ncifcrf.gov/summary.jsp] [67] was used for gene ontology (GO) [68] and KEGG (Kyoto Encyclopedia of Genes and Genomes) [69] pathway analysis. Because some genes in the bovine genome do not have known function, the GO analysis was performed with the orthologous human genes of these bovine genes. As a result, we found that the functions of these genes are enriched in multiple categories of molecular functions, including sensory perception activity, regulation of biosynthetic process and cellular processes. Some genes in common GO terms among mammals (human, mouse) were also observed in cattle, *e.g.*, the olfactory receptors gene families [13], [70], [71], [72], [73]. Besides, the KEGG analysis revealed a significant pathway, i.e., the Notch signaling pathway, which has been demonstrated to be very important in cell development in human and mouse [74], [75].We also compared the genes in the CNVRs detected in this study with those harbored in CNVRs of the human genome. As a result, 59 genes in CNVRs in the cattle genome also exist in CNVRs in the human genome (see Table S6).

In order to confirm these potential CNVRs, we performed quantitative PCR for 12 randomly selected CNVs, of which 6 were identified by two or three programs and 6 detected only by one program (PennCNV). From the former, 4 were confirmed successfully. This is similar to some previous reports in animals [21], [24]. From the later, only 2 were confirmed successfully. This suggests that using multiple CNV detection algorithms simultaneously can reduce the false positives, but it can also lead to some false negative results. It should be pointed out that those CNVs which are not confirmed by qPCR may not be really false positive. Three potential factors may contribute to this: First, SNP probes on the BovineSNP50 platform are neither dense enough nor uniformly distributed to achieve an unbiased CNVR map. Second, it is difficult to establish the exact boundaries of CNVRs. The breakpoint estimation of a CNVR may not be correct, leading to the designed primers outside the structural polymorphic region. Finally, the true CNVR boundaries may be also diverse among different animals.

In summary, we identified 99 CNVRs in Chinese Holstein by three different programs based on whole genome SNP genotyping data. These CNVRs covered 26 autosomes. Six of them were validated by qPCR successfully. Although the number of detected CNVRs here is probably an underestimate given the wide interval between SNPs in the Bovine 50 K BeadChip, the results provide a more comprehensive map of copy number variation in the cattle genome and it is an important resource for investigation of genome structure and cattle disease in the future studies.

## Supporting Information

Figure S1 **The file contains one figure with six subfigures.** The figures display the detailed information of outcomes of qPCR validation for 6 detected CNVRs.
(DOC)

Table S1 **The detailed feature of each CNVR identified in this study.**
(XLS)

Table S2 **Annotation of genes in CNVRs detected in this study.**
(XLS)

Table S3 **Information of the validated CNVRs or CNVs and the primers used in quantitative PCR analyses.**
(XLS)

Table S4 **Annotation of QTLs harbored within or partially overlapped with identified CNVRs across the bovine genome.**
(XLS)

Table S5 **High Conservation Elements (HCE) in CNVRs detected in this study.**
(XLS)

Table S6 **Gene content in the CNVRs and comparison with genes involved in Human Database of Genomic Variants.**
(XLS)

Table S7 **The proportion of total CNVRs length on each chromosome.**
(XLS)
