# Supplementary material for: Genome-Wide Identification of Copy Number Variations in Chinese Holstein
Source: PLoS One. 2012 Nov 7;7(11):e48732. doi: 10.1371/journal.pone.0048732 (PMC3492429; doi:10.1371/journal.pone.0048732)
Supplement: Figure S1 — The file contains one figure with six subfigures. The figures display the detailed information of outcomes of qPCR validation for 6 detected CNVRs. (DOC) [file pone.0048732.s001.doc]

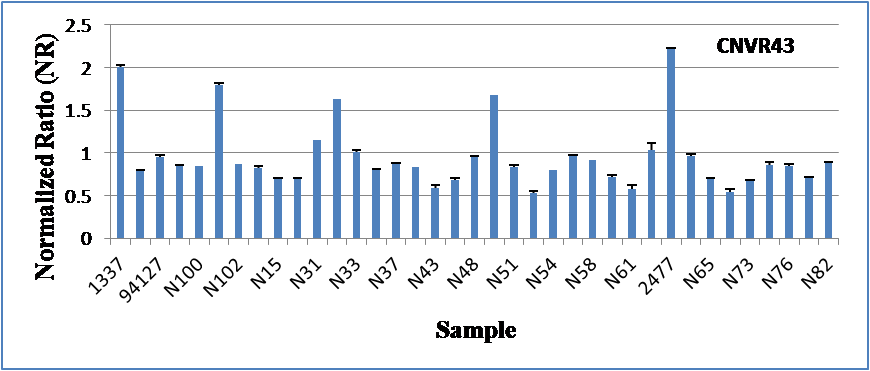


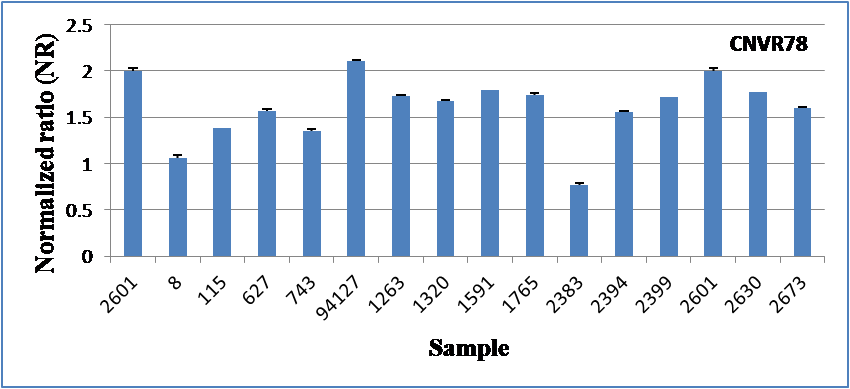


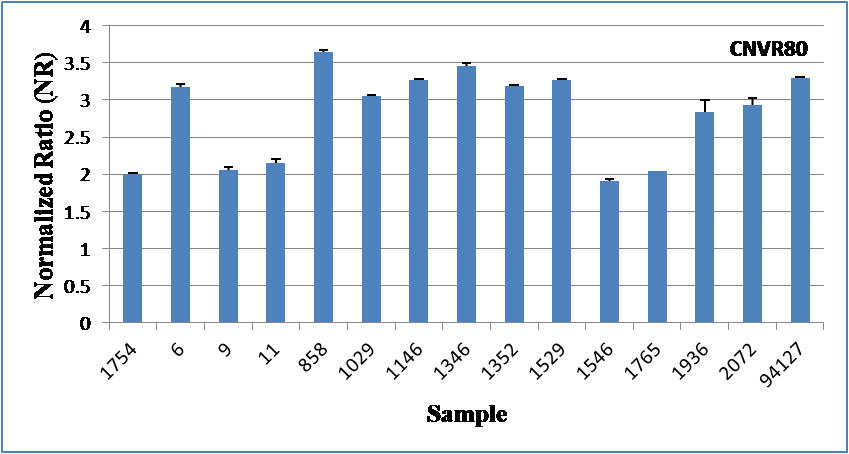


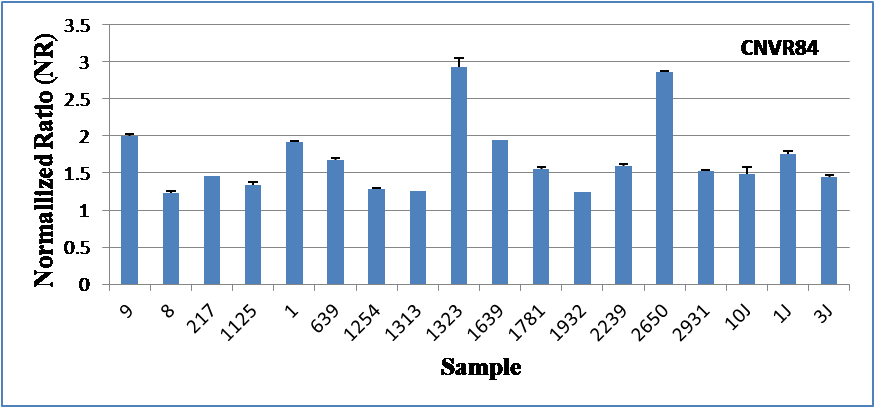


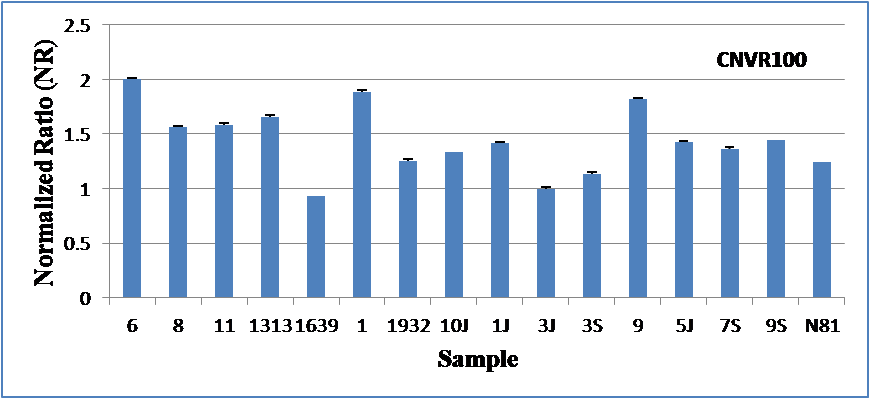


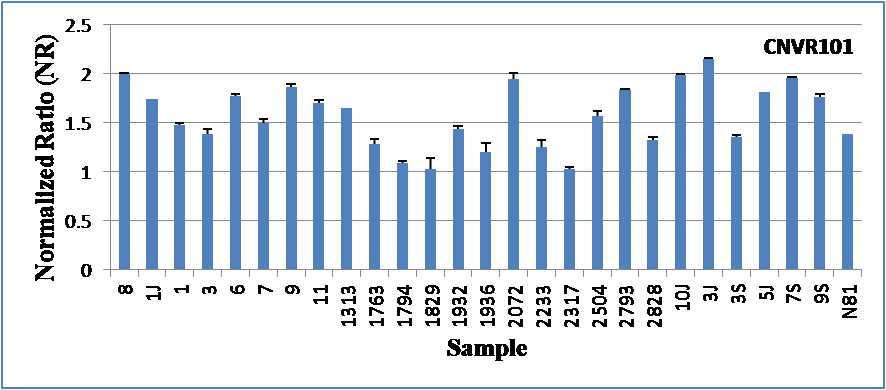


Figure S1. Results of qPCR validation for 6 CNVRs (IDs = 43, 78, 80, 84, 100, 101). NR around 2 indicates normal status (no CNV), NR around 1 indicates one copy loss, and NR around 3 or above indicates denote one or more copy gain. The error bars represent the standard error among three technical replicates.
